# Supplementary material for: Enhanced Anticancer Activity of Hymenocardia acida Stem Bark Extract Loaded into PLGA Nanoparticles
Source: Pharmaceuticals (Basel). 2022 Apr 26;15(5):535. doi: 10.3390/ph15050535 (PMC9147688; doi:10.3390/ph15050535)
Supplement: Supplementary file 1 [file pharmaceuticals-15-00535-s001.zip › pharmaceuticals-1658898-supplementary.pdf]

Enhanced Anticancer Activity of *Hymenocardia acida* Stem Bark Extract Loaded into PLGA Nanoparticles

Oluwasegun Adedokun [1], Epole N. Ntungwe [2,3], Cláudia Viegas [4,5,6], Bunyamin Adesina Ayinde [7], Luciano Barboni [8], Filippo Maggi [9], Lucilia Saraiva [10], Patrícia Rijo [2,11,*] and Pedro Fonte [4,5,12,13,*]

1 Department of Pharmacognosy, Igbinedion University, Benin 23401, Nigeria; adedokun.oluwasegun@iuokada.edu.ng
2 Research Center for Biosciences & Health Technologies (CBIOS), Universidade Lusófona de Humanidades e Tecnologias, 1749-024 Lisboa, Portugal; epole.ntungwe@ulusofona.pt
3 Department of Biomedical Sciences, Faculty of Pharmacy, University of Alcalá de Henares, 28805 Alcalá de Henares, Spain
4 Department of Chemistry and Pharmacy, Faculty of Sciences and Technology, University of Algarve, Gambelas Campus, 8005-139 Faro, Portugal; viegas.claudiasofia@gmail.com
5 Center for Marine Sciences (CCMAR), University of Algarve, 8005-139 Faro, Portugal
6 Faculty of Medicine and Biomedical Sciences (FMCB), University of Algarve, 8005-139 Faro, Portugal
7 Department of Pharmacognosy, University of Benin, Benin 23401, Nigeria; baayinde@uniben.edu
8 School of Science and Technology, Chemistry Division, University of Camerino, 62032 Camerino, Italy; luciano.barboni@unicam.it
9 Chemistry Interdisciplinary Project (ChIP), School of Pharmacy, University of Camerino, 62032 Camerino, Italy; filippo.maggi@unicam.it
10 LAQV/REQUIMTE, Laboratório de Microbiologia, Departamento de Ciências Biológicas, Faculdade de Farmácia, Universidade do Porto, 4050-313 Porto, Portugal; lucilia.saraiva@ff.up.pt
11 Instituto de Investigação do Medicamento (iMed.ULisboa), Faculdade de Farmácia, Universidade de Lisboa, 1649-003 Lisboa, Portugal
12 iBB-Institute for Bioengineering and Biosciences, Department of Bioengineering, Instituto, Superior Técnico, Universidade de Lisboa, 1049-001 Lisboa, Portugal
13 Associate Laboratory i4HB–Institute for Health and Bioeconomy at Instituto Superior Técnico, Universidade de Lisboa, Av. Rovisco Pais, 1049-001 Lisboa, Portugal
* Correspondence: patricia.rijo@ulusofona.pt (P.R.); prfonte@ualg.pt (P.F.)

Structure elucidation of Lupeol

1D- and 2D-NMR experiments of Lupeol were carried out, the following NMR experimental spectra were obtained as shown in Figures S1–S6

^{1}H NMR ($CDCl_3$, 600MHz) spectrum of Lupeol with the following spectra data: δ 4.71, 4.59 (2H, s, H-29a, 29b), 3.22 (1H, m, H-3), 2.39 (1H, m, H-19β) 1.71, 0.99, 0.85, 0.99, 0.98, 0.97, 0.90 (each 3H, s); ^{13}C NMR ($CDCl_3$, 600MHz): δ 151.0 (C-20), 109.0 (C-29), 79.0 (C-3), 55.5 (C-5), 50.5 (C-9), 48.3 (C-18), 48.0 (C-19), 43.0 (C-17), 42.9 (C-14), 40.9 (C-8), 40.0 (C-22), 38.9 (C-4), 38.7(C-1), 38.1(C-13), 37.2(C-10), 35.5(C-16), 34.2(C-7), 29.9(C-21), 28.0(C-23), 27.4(C-2), 27.1(C-15), 25.2(C-12), 21.0(C-11), 19.5(C-30), 18.5(C-6), 18.0(C-28), 16.1(C-25), 16.0(C-26), 15.5(C-24), 14.8(C-27).

The ^{1}H NMR spectrum indicated the presence of seven tertiary methyl protons at positions δ 1.71, 0.99, 0.85, 0.99, 0.98, 0.97, 0.90 (integrated for 3H-each). A sextet of one proton at δ 2.37 ascribable to 19β –H, a major characteristic of lupeol (the down shield characteristics of the peak was due to the electronegativity influence of oxygen atom on the proton). The H-3 proton showed a multiplet at δ 3.22, a pair of broad singlets at δ 4.59 and δ 4.71 (1H, each) indicated the presence of olefinic protons at (H-29a and H-29b). In addition, the double bond or pie bond effect resulted in the downfield position of the two peaks at δ 4.59 and δ 4.71, respectively. Findings from these assignments are in agreement with that reported by Jain and Bari [38].

The ^{13}C NMR experiments (Figure 3.20) indicated seven methyl groups at [δc: 28.0 (C-23), 18.0 (C-28), 16.1 (C-25), 16.0 (C-26), 15.5 (C24), 14.8 (C-27) and 19.5 (C-30)]; the signals due to an exomethylene group at [δc: 109.3 (C-29) and 151.0 (C-20)]. However, deshielded effect experienced by the signal at δc 79.0 (C-3) was a result of a hydroxyl group attached to C-3. Furthermore, the continuation of the characterisation of the structure of Lupeol was done through the 2D-NMR experiments (HMBC and COSY). In the HMBC spectrum (Figure 5), the methine proton signal at δH 3.2 (H-3) indicated cross-peaks with a methyl carbon signal (δc 28.0, C-23) by J2 correlation and a methyl carbon signal (δc 18.5, C-6) by J3 correlation. The sextet methyl signal at δH 2.37 (H-19) indicated cross-peaks with two methylene carbon signals δc 29.9 (C-21) and δc 109.0 (C-29)], a methine carbon signal [δc 48.3 (C- 18), a methyl carbon signal [δc 19.5 (C-30)] and a quaternary carbon signal [δc 151.0 (C-20)]. The pair of broad singlets of olefinic proton at δH 4.55 and 4.70 indicated cross-peaks with a methylene carbon signal [δc 48.0 (C-19) and δc 19.5 (C-30)] by J3 correlation. The COSY spectrum of BF3B2A (Figure 3.21) exhibited some cross-peaks such as between δH 2.37, H-19 and one Sp3 methylene proton signal (δH 1.37, H-21) and another Sp3methine proton signal (δ H 1.89, H18); and between oxygenated methine proton signal (δH 3.2, H-30 and Sp3 methylene signal (δH 1.60, H-2)

Based on the spectral data analysis obtained and comparison with literature, the isolated compound was suggested to be **LUPEOL (3β- lup-20(29)-en-3ol)**, a colourless pentacylic tri-terpenoid with melting point 212 – 214 ºC.

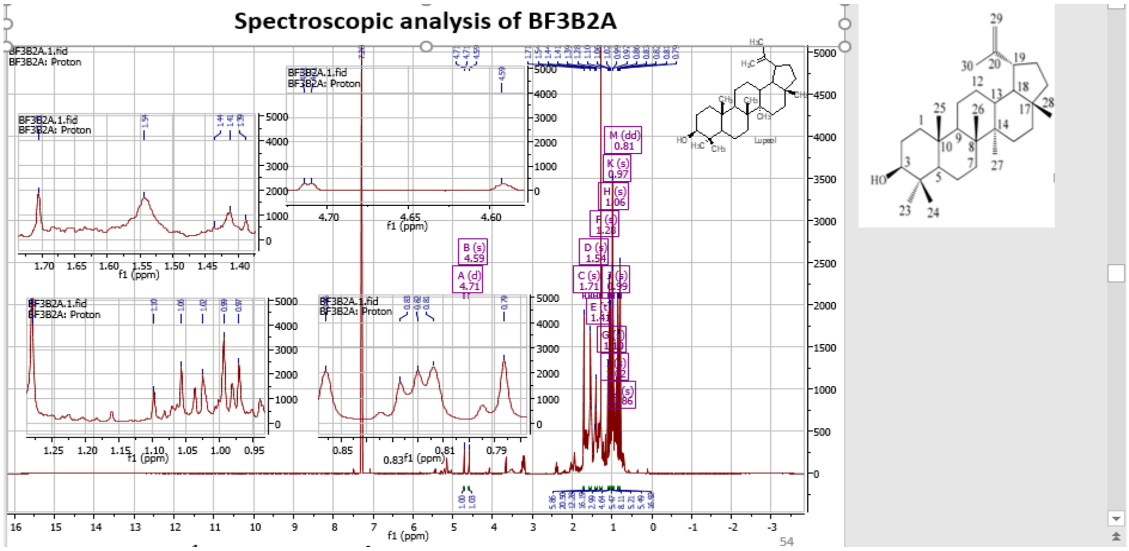

Figure S1 ^{1}H NMR spectrum of Lupeol

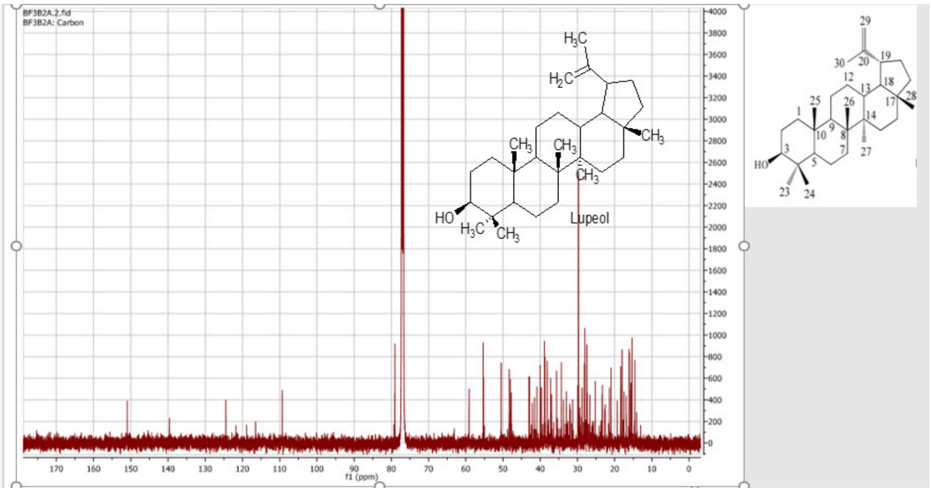

Figure S2. ^{13}C spectra of Lupeol

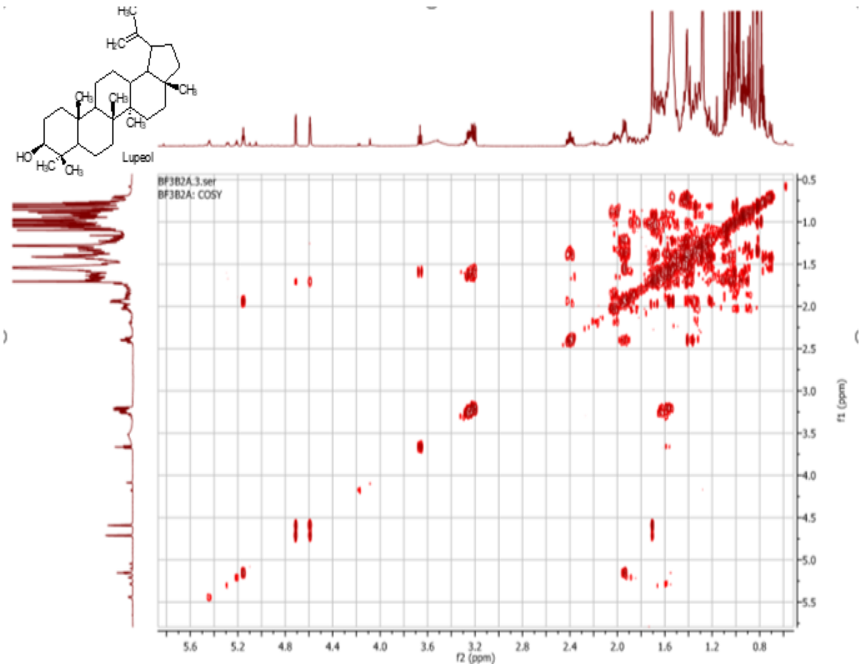

Figure S3 HSQC spectrum of Lupeol

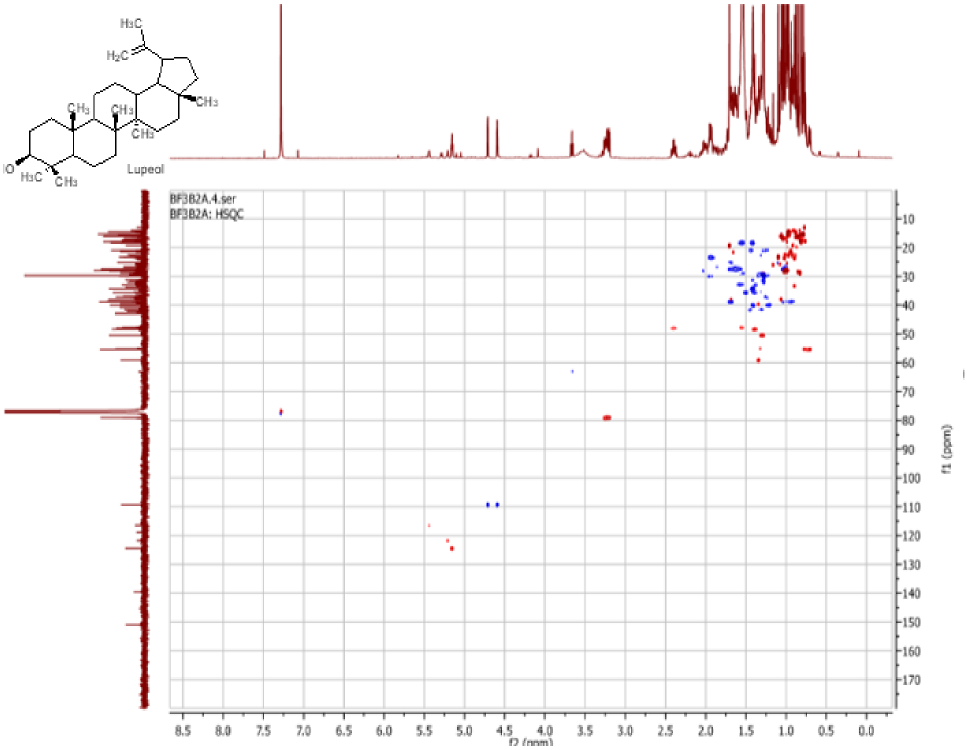

Figure S4. COSY spectrum of Lupeol

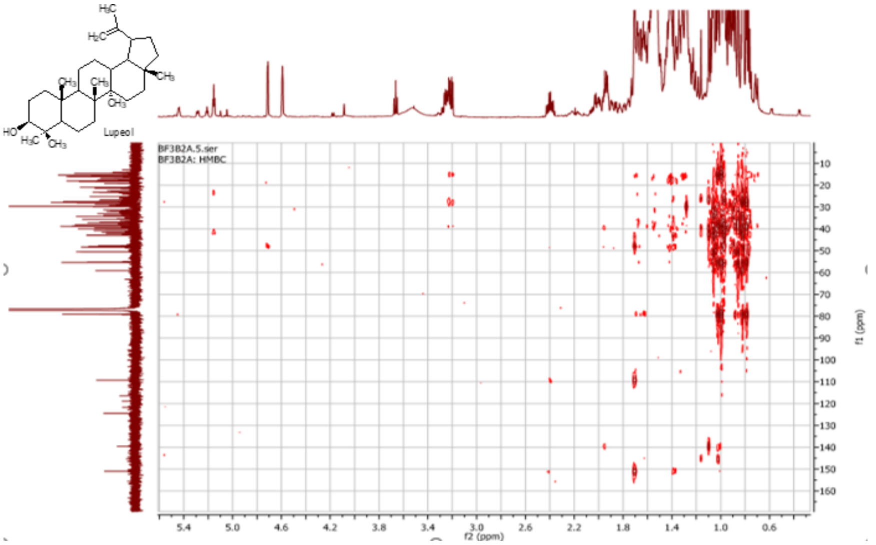

Figure S5. HMBC spectrum of Lupeol

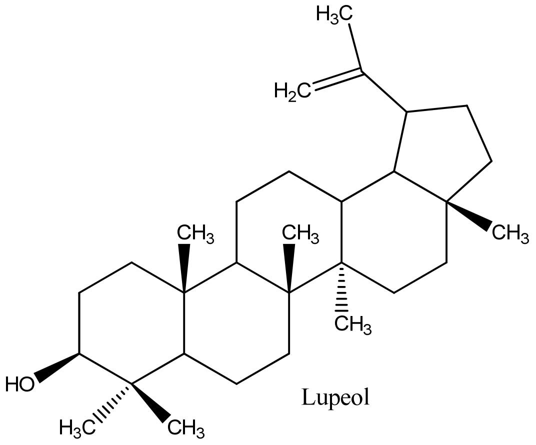

Figure S6 Compound isolated from *H. acida* suggested to be 3β- lup-20(29)-en-3ol (Lupeol)

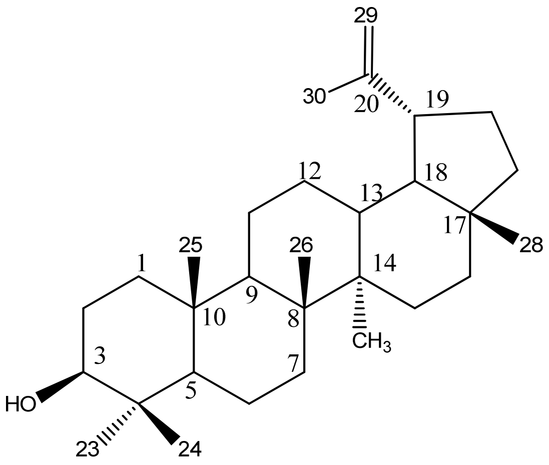

Figure S7 Numbering and melting point of Lupeol isolated from *H. acida*
